# Improved detection of DNA *Schistosoma haematobium* from eggs extracted by bead beating in urine

**DOI:** 10.1007/s00436-018-6137-7

**Published:** 2018-11-12

**Authors:** Elena Pomari, Francesca Perandin, Giulia La Marca, Zeno Bisoffi

**Affiliations:** 1 Centro per le Malattie Tropicali, IRCCS Ospedale Sacro Cuore Don Calabria, Via Sempreboni 5, I-37024 Negrar, VR Italy; 2 Department of Diagnostics and Public Health, University of Verona, Verona, Italy

**Keywords:** *Schistosoma haematobium*, Real-time PCR, DNA, Bead beating

## Abstract

Diagnosis of *Schistosoma haematobium* relies primarily on microscopical analysis of urine. The method is time consuming and requires some expertise. Genus-specific real-time PCRs have been developed, but we still observed low sensitivity. In the present study, in order to achieve a more sensitive DNA detection of eggs of *S. haematobium* in urine samples, we wanted to develop a novel protocol of DNA extraction using mechanic disruption of eggs by bead beating as supplementary step. We tested *Schistosoma* spp. internal transcribed spacer 2 real-time PCR after both methods with and without bead beating. First, we preliminary assessed the DNA detection after bead beating using dilution of 2, 10, 50, and 90 eggs/10 mL, and the Ct value analysis showed significant improved DNA detection per each point of egg concentration using the novel supplementary step. Twenty microscopy positive and five microscopy negative urine samples were used to validate the procedure. All urines came from imported cases and admitted at center for tropical medicine, and were examined by microscopy. PCR results after novel method with bead beating showed 100% to be positive for *S. haematobium*, compared with 85% positive by our standard extraction procedure. Results confirmed mechanic disruption of eggs by bead beating before DNA extraction to be highly effective method for the detection of *S. haematobium* DNA in urine.

**Electronic supplementary material:**

The online version of this article (10.1007/s00436-018-6137-7) contains supplementary material, which is available to authorized users.

## Introduction

Schistosomiasis, one of the most important neglected tropical diseases, is caused by parasitic trematode worms of the genus *Schistosoma*. Three species are of particular relevance: *S. haematobium* (causing urogenital schistosomiasis), *S. mansoni*, and *S. japonicum* (causing intestinal schistosomiasis) (Negussu et al. 2017). Almost 240 million people are infected worldwide and about 700 million people are at risk of this infection (Steinmann et al. 2006). Schistosomiasis from *S. haematobium* may remain asymptomatic for years, or just cause an often neglected, transient hematuria, before causing irreversible urogenital complications or even bladder cancer. A proper diagnosis is thus essential during the chronic phase, in order to prevent such severe problems.

Microscopic detection of *Schistosoma* eggs in stool and/or urine remains the gold standard for diagnosis (Sady et al. 2015). However, this method has low sensitivity (40–60%), in relation with the intensity of the infection, the number of samples collected and the circadian and day to day variation of egg counts (Aryeetey et al. 2013; Pillay et al. 2014). The PCR technology demonstrated to be a worthy alternative to microscopy-based diagnostic methods, particularly real-time PCR, with a high sensitivity and specificity (Verweij 2014). Various *Schistosoma* PCRs have been described (Cnops et al. 2013; Obeng et al. 2008; Sady et al. 2015; Vinkeles Melchers et al. 2014). From 2015, we adopted a real-time PCR using a set of probe/primers specific for internal transcribed spacer 2 (ITS2) *Schistosoma* spp. (Obeng et al. 2008). However, we have disappointingly observed a low sensitivity of the real-time PCR that resulted positive only in about half of microscopic-confirmed *S. haematobium* infections. In order to resolve this issue and to optimize our routine molecular testing, we focused our attention on recent pieces of evidence showing improvement in DNA extraction from intestinal parasites in fecal samples using the bead beating (Kaisar et al. 2017; Liu et al. 2013; Llewellyn et al. 2016). We hypothesized that mechanic disruption of eggs by bead beating might improve DNA extraction and detection from urinary samples. The purpose of the present study was to develop a novel protocol of DNA extraction using bead beating in order to improve the DNA detection of *S. haematobium* eggs in urine.

## Methods

The competent ethics committee (Comitato Etico for Clinical Research of Verona and Rovigo Provinces) approved this study (no. 25309) in April 2018. All included patients (*n* = 25) signed an informed consent form for the donation of their biological samples for research purpose at our center. All patients included in this study were migrants from African endemic areas. Parasitological diagnosis was performed using standard microscopy on unpreserved urine samples collected in 120-mL container between 11:00 am and 12:00 pm when *Schistosoma* eggs excretion is known to be highest (Aryeetey et al. 2013; Pillay et al. 2014). A 9-mL aliquot of each sample was centrifuged for 5 min at 3000 rpm. Next, two aliquots (200 μL) of the mixed pellet were collected; one was transferred into a 2-ml screw-capped tube without beads and one into a 2-ml screw-capped tube with ceramic beads (Roche), and frozen at − 20 °C (Kenguele et al. 2014). For microscopy, the remaining milliliter of urine was examined by the urine filtration (25-mm diameter and 12-μm pore size filter, Whatman Nuclepore), and results were expressed as corrected number of eggs/10 mL for the volume examined. Egg counts were categorized into no infection (no eggs/10 mL), low (1 < 50 eggs/10 mL), and high (≥ 50 eggs/10 mL) infection intensities (Table 1).

**Table 1:** Comparison of mean cycle threshold (Ct) and standard deviation (SD) values between sample preparation procedures with or without bead beating

*N* eggs/10 mL (microscopy counts)	*N* samples	Ct A_PCR vs Ct B_PCR mean (± SD)	*P* value
0	5	NV vs NV	NA
1–2	3	NV vs 28 (± 3.60)	NA
1–2	6	33 (± 4.02) vs 30 (± 3.53)	ns
5–49	8	31 (± 3.51) vs 25 (± 1.90)	0.0007
≥ 50	3	29 (± 1.76) vs 22 (± 1.15)	0.0049

A_PCR, PCR resulted from directly frozen sample; B_PCR, PCR resulted from bead beating supplemented on frozen sample; *NV*, negative value; *NA*, not applicable. Unpaired parametric two-tailed Student’s *t* test (GraphPad Prism v7.02) was performed, and the *P* value ≤ 0.050 was considered significant; *ns*, not significant

For each sample, the extraction of DNA was performed by both procedures: Procedure A as conventional method without bead beating and Procedure B as new method with bead beating (more details of procedures are described in Supplementary material). In particular, for Procedure B, eggs were disrupted by a beating process for 30 s at 3000 rpm using ceramic beads and a homogenization instrument (MagNA Lyser, Roche). For both procedures A and B, the DNA was extracted using MagnaPureLC.2 instrument (Roche), following the protocol DNA I Blood Cells High performance II, using the DNA isolation kit I (Roche) with a final elution volume of 100 μL.

Then, each DNA specimen extracted was analyzed by the *Schistosoma* spp. ITS2 Taqman real-time PCR (Obeng et al. 2008). The reactions, detection, and data analysis were performed with the CFX96 detection system (BioRad). The PCR reactions were considered negative/non-detected if the Ct value exceeded 40. Each run contained one negative (no DNA) and one *S. haematobium* DNA positive control. Each sample was analyzed in triplicate. As internal control for PCR inhibitors and amplification quality, the Phocine Herpes Virus type-1 (PhHV-1) DNA was amplified with specific primers/probe set as multiplex PCR (Niesters 2002).

## Results and discussion

We preliminary assessed the DNA detection after bead beating using dilution of 2, 10, 50, and 90 eggs/10 mL of 3 urine samples with 131, 113, and 95 eggs/10 mL starting counts. For each sample of dilution, the extraction of DNA was performed by both procedures: Procedure A as conventional method without bead beating and Procedure B as new method with bead beating. The quality of DNA was tested by NanoVue Spectrophotometer (GE Healthcare) and good quality with A260/A280 ratio of 1.8–2.0 was obtained in all samples processed by both procedures A and B.

**Fig. 1:**
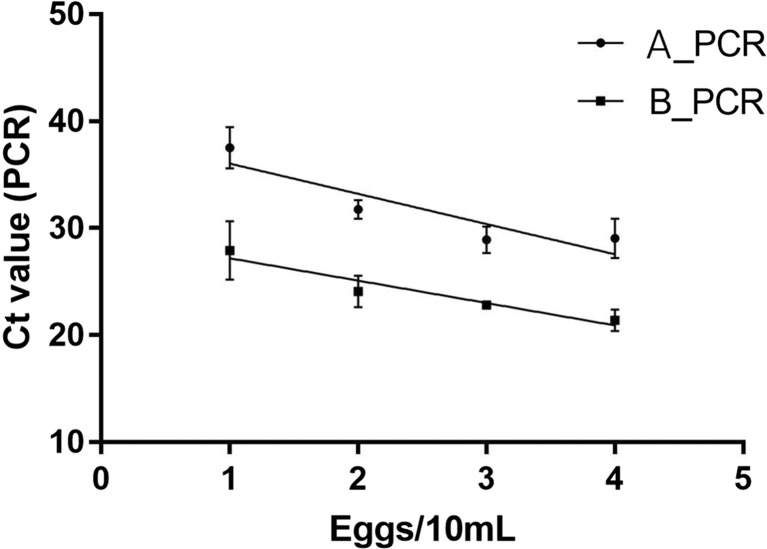
Real-time PCR results and Ct values for evaluating the sensitivity with and without bead beating for DNA extraction from *S. haematobium* eggs in urine. Increasing number of eggs/10 mL was used: 2, 10, 50, and 90. Experiments were performed in triplicate and PCR results are expressed as mean of Ct values (GraphPad Prism v7.02). Each egg sample was processed by both procedures: A_PCR, directly frozen sample; B_PCR, bead beating supplemented on frozen sample

Then, each DNA specimen extracted from this preliminary assessment was analyzed by the *Schistosoma* spp. ITS2 Taqman real-time PCR (Obeng et al. 2008). As internal control for PCR inhibitors and amplification quality, the PhHV-1 analysis showed the expected Ct ≤ 32 and the amplification of each sample was considered not hampered by inhibitory factors. The Ct values were plotted against the starting eggs equivalent per reaction by performing linear regression analysis, and *R*^2^ of 0.92 and 0.82 was obtained from real-time PCR assay after extraction of DNA with and without bead beating, respectively (Fig. 1). Results showed the goodness of data fit of PCR assays for both procedures A and B, suggesting that the DNA detection becomes more effective independently from the PCR assay performance. Indeed, we calculated ∆Ct (Ct_Procedure A–Ct_Procedure B) per each point of egg concentration and we obtained ∆Ct 9, 8, 6, and 8 respectively for 2, 10, 50, and 90 eggs/10 mL, confirming the increase of DNA detection after bead beating.

**Fig. 2:**
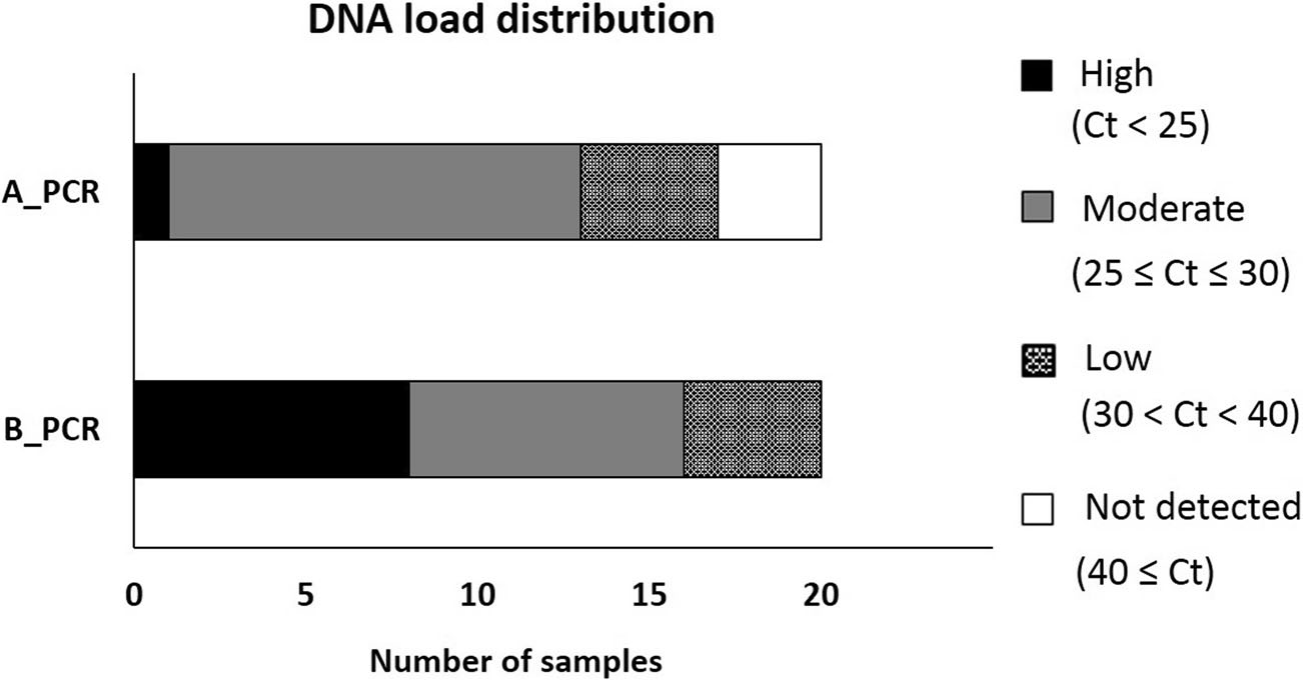
DNA load distribution of *n* = 20 *S. haematobium* egg samples using two different preparation procedures A and B on urine samples. PCR results following the sample preparation procedure: A_PCR, directly frozen sample; B_PCR, bead beating supplemented on frozen sample

Based on these results, we extended the comparison of DNA extraction with and without bead beating on 20 undiluted microscopy positive urine samples. We used 5 urine samples with no eggs detected at microscopy as true negative. For DNA extraction and real-time PCR, we followed identical conditions of procedures A and B as defined for the preliminary data described above. Among the 20 positive samples, we observed 5% high DNA load values (Ct < 25), 60% moderate (25 ≤ Ct ≤ 30), 20% low (30 < Ct < 40), and 15% not detected using the procedure A, while the procedure B showed 40% high, 40% moderate, and 20% low DNA load values, as shown in Fig. 2. Of note, only the procedure B provided 100% positivity. Table 1 reports the comparison between the median Ct values of the procedures with and without bead beating. The supplementary step of bead beating in procedure B showed a significant improved DNA detection in all samples analyzed. Moreover, the TaqMan assay resulted in specific detection of *Schistosoma* without any false-positive results, as shown by no DNA detection as expected on 5 negative urine samples. Similarly, negative controls (no DNA) provided no detection in each run (data not shown).

Schistosomiasis, mainly from *S. mansoni* and *S. haematobium*, is observed in non-endemic countries with increasing frequency. In Italy, the recent wave of asylum seekers from highly endemic areas has caused a “hidden epidemic” (Beltrame et al. 2017), with thousands of cases mostly undiagnosed, estimated to be currently living in the country. The sensitivity of direct microscopic examination as well as that of most antibody and antigen detection methods (such as CCA and CAA, mainly used for *S. mansoni* diagnosis) is unsatisfactory, as conventional PCR or real-time PCR, probably due to a lower average parasitic load if compared with endemic countries. The present findings suggest that a bead-beating procedure prior to DNA extraction has the potential to greatly increase *S. haematobium* DNA yield from urine. For the first assessment of this method, we used a limited sample size; a large-scale, prospective cohort study may provide more conclusive results from this method before indicating its routine use for the screening and diagnosis of imported urinary schistosomiasis. It would also be advisable that laboratories participate in an external quality assessment scheme, preferably a scheme using a proficiency panel of genuine clinical samples, to find out whether their DNA isolation procedure is sufficiently efficient.

## Electronic supplementary material

ESM 1 (DOCX 13.7 kb)
