# Supplementary material for: Improved detection of DNA Schistosoma haematobium from eggs extracted by bead beating in urine
Source: Parasitol Res. 2018 Nov 12;118(2):683–6. doi: 10.1007/s00436-018-6137-7 (PMC6349810; doi:10.1007/s00436-018-6137-7)
Supplement: Supplementary file 1 — (DOCX 13.7 kb) [file 436_2018_6137_MOESM1_ESM.docx]

**Supplementary material**

**DNA extraction**

The DNA will be extracted from *Schistosoma haematobium* eggs. Eggs were washed with PBS1X. The extraction of nucleic acid will be performed by two procedures: **Procedure A**: conventional method without bead-beating, and **Procedure B**: new method with bead beating; as following:

**Procedure A (conventional method without bead-beating)**

1. Prior to start the DNA extraction, all the samples (*Schistosoma haematobium* eggs) will be frozen at -20°C O/N
2. Then, samples will be thawed and added with lysis buffer (accordingly to instructions of manual)
3. Samples will be boiled for 10 min at 100 ̊C
4. Samples will be centrifuged at 10000x*g* for 1 min

**Procedure B (new method with bead-beating)**

1. Prior to start the DNA extraction, all the samples (*Schistosoma haematobium* eggs) will be frozen at -20°C O/N
2. Then, samples will be thawed and added with lysis buffer (accordingly to instructions of manual)
3. Samples will be disrupted **using bead-beating homogenizer** (MagNA Lyser Green Beads and Instrument, Roche) at 3000 rpm for 30 sec
4. Samples will be boiled for 10 min at 100 ̊C
5. Samples will be centrifuged at 10000x*g* for 1 min

For both procedures A and B, the DNA will be extracted using MagnaPureLC.2 instrument (Roche Diagnostic), following the protocol DNA_I_Blood_Cells_High performance_II, using the DNA isolation kit I (Roche) with a final elution volume of 100µl. DNA samples will be stored at -20°C until further PCR analysis.
